# Experience Sampling to Assess Burnout in Emergency Medicine: An Acceptability and Feasibility Pilot

**DOI:** 10.5811/westjem.39651

**Published:** 2025-07-18

**Authors:** Joshua J. Baugh, Justin Margolin, Ali S. Raja, Benjamin A. White

**Affiliations:** * Massachusetts General Hospital, Department of Emergency Medicine, Boston, Massachusetts; † Harvard Medical School, Department of Emergency Medicine, Boston, Massachusetts

## Abstract

**Introduction:**

Despite prior efforts to improve well-being in emergency medicine, clinician burnout in the specialty is rising. In this study we examined the acceptability and feasibility of using a method called “experience sampling” to explore factors important to clinician experience in emergency departments (ED). Experience sampling enables the measuring of work experience in real time, with more granular detail than in usual burnout surveys. The approach may reveal new opportunities for improving work experience in emergency medicine at a critical time.

**Methods:**

We conducted this pilot study in a large, urban, academic, quaternary care ED. Iterative multidisciplinary focus groups were used to generate a brief, experience-sampling tool that was comprised of three different surveys to assess emergency clinician experience before, during, and after shifts. These were deployed using a smartphone application to a convenience sample of 11 clinicians (three attending physicians, two residents, five physician assistants, and one registered nurse) during four shifts each. A post-pilot survey was also sent to all participants to evaluate their experience of using the tool. Our primary outcome measures were feasibility, assessed by the survey response rates during the pilot, and acceptability, assessed by participant sentiment as expressed in the post-pilot surveys. Secondary outcomes were quantitative- and qualitative-experience data collected using the tool.

**Results:**

The overall response rates for pre-shift, on-shift, and post-shift surveys were 79%, 73%, and 91%, respectively. All participants responded to the post-pilot survey and indicated they would be willing to use the experience-sampling tool again in the future. Many participants noted that the simple and open-ended on-shift questions were relatively easy to complete; some also said on-shift survey questions could present added difficulty during busy shifts. Four participants said the exercise of completing surveys itself improved on-shift experience by prompting reflection. Common themes associated with positive experiences included manageable patient volumes, excellent teamwork, interesting cases, adequate staffing, and feeling able to provide adequate care. Common themes associated with negative experiences included crowding, inadequate staffing, feeling overwhelmed, complex patient cases, difficult disposition plans, and feeling unable to provide adequate care.

**Conclusion:**

Experience sampling is an acceptable and feasible method for measuring clinician experience in a busy academic ED. Further studies could potentially use this approach to identify targets for reducing burnout in emergency medicine.

## INTRODUCTION

Burnout among healthcare professionals is a threat to care quality, healthcare costs, and the availability of a robust healthcare workforce to serve the public.^1^ Over 50% of physicians nationwide are experiencing professional burnout, with even higher numbers in other healthcare roles.^2^,^3^ Amidst increasing well-being concerns broadly, emergency medicine (EM) is an outlier, with the highest physician burnout rate of all specialties.^4^–^6^ There was a large increase in burnout among emergency physicians between 2021 and 2022—from 43% to 60%—and emergency physician burnout remains above 60% as of 2024.^7^ The escalating prevalence of burnout poses significant challenges to the future of the EM workforce.

As defined by Maslach, burnout is the triad of depersonalization, emotional exhaustion, and a decreased sense of personal accomplishment.^8^ While traditional approaches to addressing burnout have largely focused on interventions aimed at individuals, mounting evidence suggests that organizational, administrative, and department-specific factors explain most of healthcare burnout.^9^,^10^ Prior literature also suggests that interventions aimed at systemic organizational features are more likely to succeed in meaningfully decreasing burnout.^11^ Despite this increasing understanding, the high EM burnout rate suggest that new approaches are needed to address the problems faced by our specialty.

Nearly all healthcare workplace-experience research to date has used surveys that ask personnel to reflect on weeks or months of work to identify features that increase or decrease work satisfaction. However, workplace-experience research in other fields has used a methodology called “experience sampling.” Workplace-experience sampling is a way of gathering data with brief, repeated assessments of current experience while individuals are actively working; it allows an analysis of hour-to-hour work experience during specific activities. Over a defined period, researchers can gather a rich set of real-time, self-reported data points that provide a granular level of detail not possible with traditional surveys and with less risk of recall bias.^12^ In other fields, experience sampling has deepened understandings of workplace experience and revealed novel areas for intervention, and one study of pediatric emergency clinicians has used a version of this methodology.^13^–^16^

It may be difficult to ask healthcare personnel to complete experience-sampling surveys in real time during busy workdays, particularly in EM where the pace of work is rapid and interruptions abound.^17^ Yet an experience-sampling approach might reveal opportunities to improve the EM work experience that have thus far been overlooked. We, therefore, undertook an acceptability and feasibility pilot study of experience sampling in an EM context.

## METHODS

### Study Setting and Design

We conducted this study in an urban, academic, quaternary-care center emergency department (ED) with approximately 120,000 patient visits per year. This ED has an EM residency program and a large physician assistant (PA) program. The study was approved by our institutional review board.

### Development of the Experience Sampling Tool

An experience sampling tool for use in the ED setting requires validated approaches to assessing workplace experience, while also being brief enough to feasibly be completed during busy shifts. Our team used an iterative, focus-group approach to develop the different survey questions, bringing together frontline clinicians and experts to review validated surveys and pare them down to a usable tool. Key topics for the groups included the following: the positive or negative language used; the suitability of free-text vs multiple-choice responses; and the frequency and length of survey questions.

**Population Health Research Capsule**

What do we already know about this issue?

*Experience sampling has revealed new insights about work experience in fields other than emergency medicine (EM)*.

What was the research question?

*Is experience sampling through a mobile phone app acceptable and feasible in a busy emergency department?*

What was the major finding of the study?

*Response rates for all on-shift, experience-sampling prompts were >70%, and participants rated their experience favorably*.

How does this improve population health?

*Experience sampling may reveal new insights for improving burnout in EM, a critical workforce issue in our field*.

The first focus group was comprised of 12 ED personnel from our institution, including attending physicians, resident physicians, PAs, and nurses. During this focus group, participants were asked to rank the utility of questions from three previously validated work experience tools: the Maslach Burnout Inventory; the Utrecht Work Engagement Scale; and the Work-Related Flow Inventory (WOLF), which led to the prioritization of question types.^8^,^18^,^19^ The group felt emergency clinicians would only have bandwidth for one quantitative question and one qualitative question for each on-shift survey question. They also believed it would be feasible to ask a longer list of follow-up questions after a shift to summarize experiences from that shift. Through iterative discussion, the group arrived at two concepts most important to encapsulate their work experience: the enjoyment of work, and pride in work.

They agreed the optimal on-shift question was a multiple-choice question, “Are you enjoying your shift?” followed by a qualitative question, “Why?” It was decided that this survey question would be delivered every two hours during shifts, balancing a desire for repeated data collection with the need not to disrupt clinical work. For the post-shift survey, the group agreed that the most important question was, “Did you feel proud of your work today?” The group also decided to include a question about burnout explicitly, as well as questions about the ability of clinicians to meet the challenges they faced on shift, derived from the WOLF. Additionally, they decided it would be important to ask a few questions before each shift to assess clinician state of mind going into work. Finally, they noted that it would be important to encourage “quick thoughts” and sentence fragments in free-text responses, so that participants would not feel pressured to write long, carefully worded answers.

After the first focus group of frontline clinicians developed the drafted survey questions, a second focus group of experts in EM operations and experience coalesced at a national EM conference to review and refine the surveys. Members of this focus group largely echoed the sentiments of the frontline clinician groups, emphasizing the need for brief questions at spaced intervals, followed by a longer post-shift survey. The second group made small modifications to precise question language but did not suggest any major changes to the questions chosen or themes explored. Given the minimal modifications made by this group of experts to the initial draft of survey questions, we decided that a third focus group was not warranted. See Table 1 for the final list of survey questions.

### Survey Delivery Platform

After exploring options and in discussion with our focus groups, we decided a mobile phone application would be the best approach to deliver real-time survey questions to clinicians. This approach would not require clinicians to carry any extra devices and would facilitate rapid, easy responses. We ultimately chose an existing mobile phone-survey delivery platform LifeData (LifeData, LLC. Marion, IN). The tool was programmed such that completion of the pre-shift survey would trigger timed notifications for the current experience (on-shift) and post-shift surveys, depending on shift length, as delineated in the pre-shift questions. For timed notifications during shifts, participants would receive a reminder if they did not answer a question after 15 minutes. If that question was not answered by the time the next timed question was sent, the participant would no longer have the option to answer the previous question.

### Participant Recruitment and Pilot Design

To recruit a cohort of pilot participants, emails were sent out to a convenience sample of clinicians across the ED. A total of 14 emergency clinicians representing various role groups (attending physicians, resident physicians, PAs, and registered nurses) were invited via email to participate in the pilot program. Potential participants were asked to use the tool during four of their ED shifts and to fill out a post-pilot survey assessing the acceptability of the experience. Three individuals declined participation as they did not have four shifts during the pilot period. Therefore, the final participating cohort of 11 clinicians was comprised of three attending physicians, two resident physicians, five PAs, and one registered nurse. Participants each received a $10 gift card.

Detailed email instructions guided participants through the downloading and registration process for the LifeData mobile application. Participants were asked to provide four shifts during which they were willing to use the tool. Thirty minutes before the start of each designated shift, a reminder email was sent to participants to complete the pre-shift survey. Participants were reminded to check their emails just prior to the start of their shift. Successful completion of the pre-shift survey subsequently triggered the current experience (on-shift) survey questions to be delivered at two-hour intervals during their shift, and a post-shift survey to be delivered 30 minutes following the shift’s completion.

### Assessing Experience with the Tool

All participants also received a post-pilot survey assessing their overall experience using the experience sampling tool. The following survey questions were asked, and for several of the following questions, participants were asked to respond on a Likert scale:

*– Which role group do you belong to? (free text)*
*– What was your experience of filling out prompts during shifts? (free text)*
*– How did you find the length of the pre-shift survey? (“too long” [1] to “could have asked more” [3])*
*– How did you find the frequency of the prompts during shifts? (“too few” [1] to “too many” [3*]*)*
*– How did you find the length of the post-shift survey? (‘too long” [1] to “could have asked more” [3])*
*– How did you feel about the questions that were asked? (free text)*
*– Are there other questions you wish we had asked? (free text)*
*– We are considering asking staff broadly to fill these out during a portion of shifts. What do you think of this idea, and what would you suggest to make the effort successful? (free text)*
*– Would you be willing to use the tool during future shifts? (yes/no)*

### Outcome Measures and Statistical Analysis

Primary outcome measures were feasibility, assessed by the survey response rates during the pilot, and acceptability, assessed via participant sentiments about using the tool expressed in the post-pilot survey. We performed simple quantitative and qualitative analyses for responses to surveys by the pilot participants. These were conducted only to explore the potential of the experience-sampling approach to generate useful data; the study was not powered to draw any conclusions from these responses. Quantitative measures were simple means and standard deviations for responses. We explored qualitative themes using an iterative coding approach, wherein one author created initial thematic codes and additional authors provided input for quotes where the theme was not immediately clear. Once thematic codes were finalized, we tabulated code frequencies.

## RESULTS

### Feasibility: Participation and Response Rates

Each of the 11 participating clinicians was asked to use the tool during four shifts, resulting in 44 possible shifts for the pilot. Of these 44 shifts, there was a response rate of 79% for the pre-shift survey. For shifts where the pre-shift survey was completed—and, therefore, the rest of the survey was triggered—the response rate for the on-shift questions was 73%, and the response rate for the post-shift survey was 91%.

### Acceptability: Post-Pilot Survey Responses

The response rate for the post-pilot survey was 100%. In response to the question: “Would you be willing to use this tool during future shifts,” 100% (11/11) of participants responded “yes.” When asked about the frequency of survey questions, all 11 participants indicated that the every-two-hour frequency was optimal. Nine of 11 participants indicated the pre-shift survey was the right length, while two said it could have been longer. Ten of 11 participants indicated that the post-shift survey was the right length, while one participant indicated it could have been longer.

Multiple themes emerged in the qualitative responses to the post-pilot surveys. Seven participants noted that the simplicity and open-ended nature of the questions made responses relatively easy. Six participants said that it was difficult to respond to on-shift survey questions during the busiest parts of their shifts. Five participants noted that the mobile application was easy to use. Four participants stated that using the tool facilitated self-reflection about their shifts in a way that was helpful for their perceptions of work experience.

### Quantitative Measures

In the pre-shift survey question that asked whether participants were looking forward to their shift, the average answer was 2.9 of 5 (SD 0.7). The current experience (on-shift) survey was sent to participants a total of four times in each shift (in two-hour intervals). The corresponding values for on-shift experience decreased over the course shifts on average. For the on-shift question of “Are you enjoying your work right now,” the following were the average values (with SD) observed for each successive question, respectively: first, 3.4 (0.9); second, 3.4 (1.0); third, 2.9 (0.9); and fourth, 2.9 (0.4). The average score for “I feel proud of my work today” was 3.3 (SD 0.6). The average for “I feel burned out by my work today” was 3.0 (SD 0.6).

There were 15 shifts where participants stated they were able to appropriately meet the challenges of the day; during 14 shifts participants noted feeling overwhelmed and unable to meet the challenges of the day, while during eight shifts, participants noted feeling bored. See Table 2 for details. Above are the mean with SD for responses to select quantitative responses from our surveys. “Looking forward to shift” reflects answers to the pre-shift survey question: “Are you looking forward to today’s shift?” (scale of 1–5). “Current experience” 1–4 reflect answers to the on-shift survey question “Are you enjoying your work right now?” (1–5) during the first, second, third, and fourth instances that it was asked during shifts. “Post-shift pride” reflects answers to the post-shift survey question: “I feel proud of my work today” (1–5). “Post-shift burnout” reflects answers to the post-shift survey question: “I feel burned out by my work today” (1–5).

### Qualitative Themes

Several themes emerged in the current experience (on-shift) and post-shift survey responses regarding both positive and negative experiences (Figure 1). The most common themes related to positive work experiences were manageable patient volume, excellent teamwork, interesting cases, adequate staffing, and an overall feeling of being able to provide adequate care. In contrast, the most common themes related to negative work experiences were high patient volumes, crowding, inadequate staffing, feeling overwhelmed, complicated patient cases, poor disposition plans, signing out late, and an inability to provide adequate care.

## DISCUSSION

Overall, the results of our pilot study suggest that experience sampling using a mobile phone application is a feasible and acceptable approach to assessing work experience for emergency clinicians. This approach may reveal ways to decrease EM burnout that have not yet been explored. Participants engaged with the tool during nearly 80% of their designated shifts, with response rates to on-shift survey questions of over 70%, and response rates to post-shift surveys of over 90%. Of the participants, 100% stated that they would be willing to use the tool in the future, and most participants felt the length and frequency of the different survey components were appropriate. The main barrier to the approach appears to be that most participants found that it could be difficult to complete survey questions during the busiest parts of shifts; this was not surprising, and the response rates observed suggest that this can be overcome. Of note, a previous study of experience sampling in a pediatric ED used in-person prompters and a much longer list of questions^16^; while that study obtained rich data and bolstered the case for using experience sampling in healthcare contexts, our mobile phone approach may provide a more sustainable strategy for ongoing experience sampling in EM contexts.

Surprisingly, four of the eleven participants noted that use of the tool itself improved their work experience by encouraging them to reflect on their shifts. This raises the possibility that the experience sampling approach might have direct benefits for participants in addition to facilitating data collection. This finding may ease trepidation about the use of such tools during clinical work, but it also must be explored in more depth to assess whether it is durable.

The experience-sampling approach allowed for the collection of rich data about work experience, identifying themes associated with positive and negative work experiences in real time, including patient volumes, crowding, acuity, staffing, teamwork, and communication. These themes reflect prior burnout research that suggests systemic features of the workplace are highly relevant to feelings about work.^10^,^20^,^21^ Interestingly, experience scores were higher during the first half of shifts, with lower scores later in shifts, which matches prior literature.^16^ This pilot sample was too small to draw any firm conclusions, but it perhaps suggests questions that might be answered with a larger experience-sampling study.

An important potential benefit of experience sampling may be the ability to relate work experience to objective environmental and temporal features of ED shifts. Participant scores could be mapped to objective ED features such as patient arrivals, acuity mix, staffing levels, crowding, and team composition in a day-to-day or even hour-to-hour fashion, allowing researchers to assess relationships between clinician perceptions and operational realities. This might allow more causal explanations for work experience trends than is generally possible using burnout surveys that encompass weeks or months of experience.

## LIMITATIONS

There were several limitations associated with this pilot study. It was conducted at a single site with a small convenience sample; larger multisite studies will be needed to confirm the feasibility of a mobile phone-based experience-sampling approach in EDs across role groups. The study was also too small to draw any conclusions about burnout from the clinician experience data itself; a larger study will be needed to answer questions about what may be most helpful for emergency clinician burnout that is not already being addressed.

## CONCLUSION

Our study suggests that experience sampling is an acceptable and feasible method to study clinician experience in emergency departments and may allow the generation of rich data that can answer questions previously difficult to examine with traditional surveys. Future work should test this approach in a larger sample as emergency medicine explores solutions for persistently high burnout in the field.

## Supplementary Information



## Figures and Tables

**Figure:**
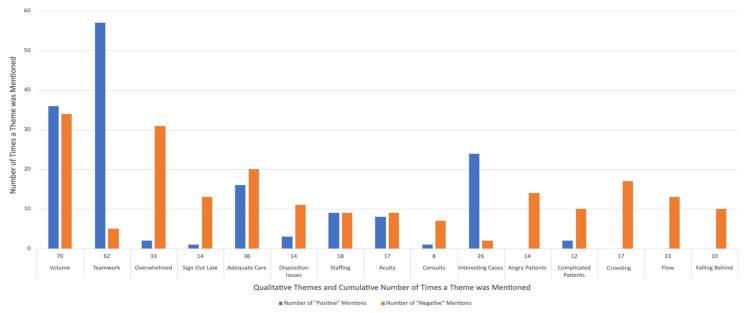
Qualitative themes and associated positive or negative valences for on-shift and post-shift survey responses using the emergency departpment experience-sampling tool.

**Table 1:** Survey questions for pre-shift, current experience, and post-shift surveys.

**Pre-shift Questions**	Which role group do you belong to?
	What area of the ED is your shift today?
	How long is your shift today?
	Are you looking forward to today’s shift? (1 to 5, 1 = not at all, 5 = very much so)
	Why or why not? (free text)
**Current Experience Questions**	Are you enjoying your work right now? (1 to 5, 1 = not at all, 5 = very much so)
	Why or why not? (free text)
**Post-shift Questions**	I feel proud of my work today (1 to 5, 1 = not at all, 5 = very much so)
	I feel burned out by my work today (1 to 5, 1 = not at all, 5 = very much so)
	How did you feel about your ability to handle what was asked of you during the shift? (1 to 5, 1= I was completely bored, 3 = I was able to meet the challenge of the day, 5 = I was completely overwhelmed)
	What contributed most to the above feeling? (free text)
	What were the worst things about your shift today? (free text)
	What were the best things about your shift today? (free text)
	What could be changed to have made this a better shift? (free text)

*ED*, emergency department.

**Table 2:** Mean scores and standard deviations (SD) for select survey questions.

Looking forward to shift		Current experience 1		Current experience 2		Current experience 3		Current experience 4		Post-shift pride		Post-shift burn out	
Mean	SD	Mean	SD	Mean	SD	Mean	SD	Mean	SD	Mean	SD	Mean	SD
2.92	0.70	3.38	0.85	3.36	1.03	2.85	0.88	2.85	0.43	3.32	0.57	2.96	0.62
