# Supplementary figures and images for: Experience Sampling to Assess Burnout in Emergency Medicine: An Acceptability and Feasibility Pilot
Source: West J Emerg Med. 2025 Jul 18;26(4):1105–11. doi: 10.5811/westjem.39651 (PMC12342573; doi:10.5811/westjem.39651)

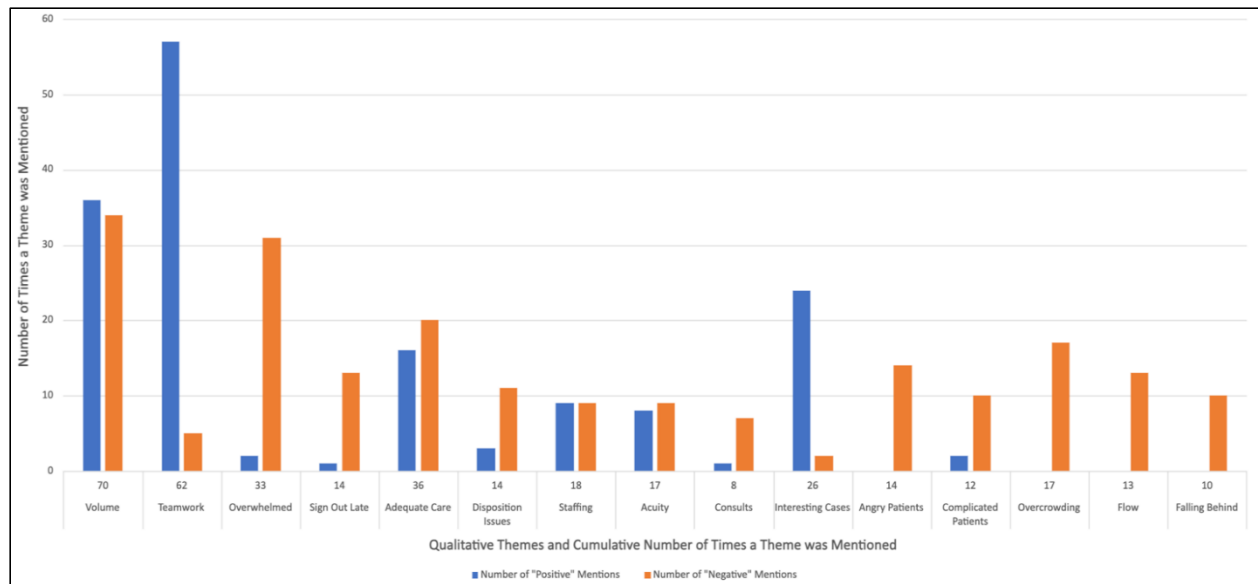

Supplement: Supplementary file 1 [file wjem-26-1105-g001.pdf]
